# Brief quiet ego contemplation reduces oxidative stress and mind-wandering

**DOI:** 10.3389/fpsyg.2015.01481

**Published:** 2015-09-30

**Authors:** Heidi A. Wayment, Ann F. Collier, Melissa Birkett, Tinna Traustadóttir, Robert E. Till

**Affiliations:** ^1^Department of Psychological Sciences, Northern Arizona University, Flagstaff, AZUSA; ^2^Department of Biological Sciences, Northern Arizona University, Flagstaff, AZUSA

**Keywords:** quiet ego, oxidative stress, SART, compassionate self-identity, brief intervention

## Abstract

Excessive self-concern increases perceptions of threat and defensiveness. In contrast, fostering a more inclusive and expanded sense of self can reduce stress and improve well-being. We developed and tested a novel brief intervention designed to strengthen a student’s compassionate self-identity, an identity that values balance and growth by reminding them of four quiet ego characteristics: detached awareness, inclusive identity, perspective taking, and growth. Students (*N* = 32) in their first semester of college who reported greater self-protective (e.g., defensive) goals in the first 2 weeks of the semester were invited to participate in the study. Volunteers were randomly assigned to one of three conditions: quiet ego contemplation (QEC), QEC with virtual reality (VR) headset (QEC-VR), and control. Participants came to the lab three times to engage in a 15-min exercise in a 30-days period. The 15-min QEC briefly described each quiet ego characteristic followed by a few minutes time to reflect on what that characteristic meant to them. Those in the QEC condition reported improved quiet ego characteristics and pluralistic thinking, decreases in a urinary marker of oxidative stress, and reduced mind-wandering on a cognitive task. Contrary to expectation, participants who wore the VR headsets while listening to the QEC demonstrated the least improvement. Results suggest that a brief intervention that reduces self-focus and strengthens a more compassionate self-view may offer an additional resource that individuals can use in their everyday lives.

## Introduction

The transition to college can be a particularly stressful time in a young person’s life (Ross et al., 1999; Dyson and Renk, 2006; Pryor et al., 2010). Stress may be influenced by many factors at this time, including inadequate time-management skills, underdeveloped study skills, interpersonal conflict, or negative experiences outside of the academic setting. Not surprisingly, between 30 and 55% of college students report greater than average levels of stress, with depression and anxiety the most common reactions to stress (Regehr et al., 2013; ACHA, 2014). A recent meta-analysis has concluded that many types of stress interventions for college students, including cognitive, behavioral, and mindfulness-based interventions, are all effective in addressing these aforementioned factors and in so doing, mitigate the effects of stress on college students (Regehr et al., 2013). Unfortunately, these interventions are often under-utilized because of the time-commitment (e.g., multiple sessions ranging from 45 min to 2 h) and the perceived stigma associated with receiving services at a campus mental health center (Regehr et al., 2013). Regardless of the underlying causes of stress, however, individuals who are overly self-focused and self-critical (e.g., the “curse of the self" Leary, 2004) may be increasing their risk for stress and minimizing their ability to cope. Indeed, the transition to college can increase self-uncertainty and give rise to increased self-evaluative activity (Wayment and Taylor, 1995). Biased thinking about the self and others (e.g., excessive self-protective, negative, or otherwise distorted) is associated with negative affect and psychological distress (Greenberg and Pyszczynski, 1986; Wood et al., 1990; Mor and Winquist, 2002; Watkins, 2008; Lukianoff and Haidt, 2015). On the other hand an excessive focus on others is also associated with depressive symptoms, lower well-being, and more negative psychosocial adjustment (Helgeson, 1994). Thus, to manage the stress associated with the transition to college, an ability to balance self- and other-focus and reduce defensive thinking should be helpful.

Thus, the purpose of this study was to test whether a brief intervention that increased the salience of values and skills supporting a balanced self-identity could have specific cognitive and physiological benefit to students making the transition to college. A balanced sense of self has psychosocial benefits (Cohen and Sherman, 2014; Critcher and Dunning, 2015). There are two literatures that support our study goals. First, the literature on self-affirmation processes demonstrates that when one’s perspective on the self is broadened (e.g., expanded), threat and defensiveness are reduced (Schimel et al., 2004; Burson et al., 2012). A closer look at the values associated with positive self-affirmation effects are those that are transcendent, those that emphasize social connections and being part of purposes or projects that go “beyond” the self (Crocker et al., 2008; Shnabel et al., 2013). Further, even very brief interventions can have lasting impacts, most notably in the area of reduced defensiveness (Critcher and Dunning, 2015), more balanced information processing (Correll et al., 2004), better working memory (Logel and Cohen, 2012), breaking ruminative cycles (Lejuez et al., 2001), and reducing physiological correlates of stress (Creswell et al., 2005; Sherman et al., 2009).

A second, literature suggests that a compassionate mindset that reflects an expanded sense of self, is increasingly recognized as an important psychosocial resource. For example, among college students making the transition to college, Crocker and Canevello (2008) and Canevello and Crocker (2011) have found that compassionate interpersonal goals positively impact academic achievement, and more rewarding personal relationships. Conversely, self-image goals, those primarily concerned with defending desired self-images, are associated with problematic and risky behavior. In a 12-weeks longitudinal study of nearly 200 first-year college students, self-image interpersonal goals were associated with increases in distress over time whereas use of compassionate goals was associated with decreased stress. In another 12-weeks study of 115 first-semester roommate pairs, the same patterns were detected in same-week, lagged-week, and pretest-to-posttest analyses, even when controlling for known risk factors of stress. Crocker and colleagues (Abelson et al., 2014) altered the instructions to a self-evaluative stressor task (TSST; Trier Social Stress Test) in a way that made compassionate interpersonal goals salient. The TSST asks participants to engage in an interview and math task designed to heighten stress. Participants exposed to the TSST stressor demonstrated lower stress reactivity to that stressor (e.g., activation of hypothalamic–pituitary–adrenal axis, HPA, as measured by salivary cortisol). Compassion-based interventions have been shown to protect mental and physical health (Klimecki et al., 2013; Roeser and Pinela, 2014). Compassionate mind training (CMT) was developed to address shame and self-criticism by promoting compassion for self and others in group therapy settings (Gilbert and Procter, 2006; Gilbert, 2009).

We designed and tested an intervention based on the concept of the “quiet ego.” Borrowing heavily from humanistic, organismic, and eudemonic perspectives on the self, Wayment and Bauer (2008) coined the term “quiet ego” in order to convey an identity that is not excessively self-focused but also not excessively other-focused—an identity that incorporates others without losing the self. The four quiet ego characteristics, described in detail elsewhere (Bauer and Wayment, 2008; Wayment et al., 2015) are: detached awareness (awareness of self- and other without judgment, mindfulness), inclusive identity (interconnection and interdependence), perspective-taking (foundation for compassion and empathy), and growth (recognition of the importance of development and growth over time). With the quieted ego, there is more balance and integration of the self and others in one’s concept of self, a balanced self-identity that facilitates personal growth and a greater compassion for the self and others (Wayment et al., under review).

Thus, the goal of this study was to examine whether reflecting on quiet ego characteristics could be helpful during the transition to college. First, we hypothesized that listening to, and reflecting on, quiet ego characteristics would make students’ compassionate goals and sense of identity more salient. We expected that our intervention would increase scores on measures capturing this ability: quiet ego and pluralistic thinking. The quiet ego scale measures quiet ego characteristics. Higher scores on this scale are associated with personality variables, cognitive style, coping styles, attitudes, and values consistent with a balanced self-identity (Wayment et al., under review) Individuals higher in pluralistic thinking perceive themselves to be relatively better at understanding divergent perspectives and being more open-minded and tolerant (Hurtado, 2001). We also expected that our intervention would be associated with decreased stress. We chose to measure stress with an objective marker: oxidative stress (Gingrich, 2005; Salim, 2014). F2-isoprostanes are stable, sensitive biomarkers of oxidative stress, specifically lipid peroxidation (Milne et al., 2005). Although levels of this biomarker tend to increase with age and are associated with chronic diseases such as cardiovascular disease and diabetes (Traustadottir et al., 2012), a recent meta-analysis of 23 studies (*n* = 4980) has found that oxidative stress may also mediate the link between depression and poor health outcomes (*d* = 0.55; Palta et al., 2014).

As mentioned earlier, mindfulness-based interventions are effective in reducing college-related stress (Regehr et al., 2013). Findings from a recent meta-analysis suggests that mindfulness training programs may be effective due to the emphasis placed on learning how to focus attention, evidenced by improvements in executive attention (Chiesa et al., 2011). Mindfulness meditation interventions have shown demonstrated improvement in an ability to sustain attention (Zeidan et al., 2010) and reduce mind-wandering (Mrazek et al., 2012a). Brief versions of mindfulness meditation training have been shown to be beneficial (Banks et al., 2015). Thus, we examined whether our brief quiet ego intervention would reduce mind-wandering in a lab setting (Mrazek et al., 2012b). We reasoned that a recording that was short might have future application in other settings such as a clinical or medical setting.

Given our interest in developing a self-management strategy that could be delivered in an audio format, we also elected to add an additional experimental condition that we reasoned might provide an even better focused experience. Thus, our second experimental condition involved having participants listen to the quiet ego contemplation (QEC) audio while wearing a virtual reality (VR) headset and having the participant immerse him/herself visually in a beautiful, park-like setting. Recent research has indicated that when VR and audio are combined, there are enhanced relaxation and positive affect effects for individuals experiencing stress and emotionally provocative situations (Riva et al., 2000, 2012; Riva, 2005; Baños et al., 2011; Villani and Riva, 2012; Ghanbarzadeh et al., 2014; Turner and Casey, 2014). In fact, the mediated, interactive experience of VR alone, in a virtual natural setting, is extremely restorative; it is associated with decreases in stress and improved affect (Valtchanov et al., 2010). There is also research that suggests “awe” experiences, frequently associated with places of profound beauty and connectedness with nature, have the ability to expand the sense of self (e.g., putting the “self” in perspective; Shiota et al., 2003). We reasoned that by combining VR with the QEC audio, there would be a synergistic effect such that we would create a more vivid and compelling intervention.

## Materials and Methods

### Participants

Participants were 32 female undergraduate students at a southwestern university who earned course credit and $100 for their participation in a study that spanned the latter half of their first semester in college. Average age was 18.16 (*SD* = 0.45). Seventy-two percent of the sample identified as white/Caucasian, 9.4% Black/African American, and 18.8% Hispanic/Latina.

### Study Design

#### Screening Procedures

**FIGURE 1:**
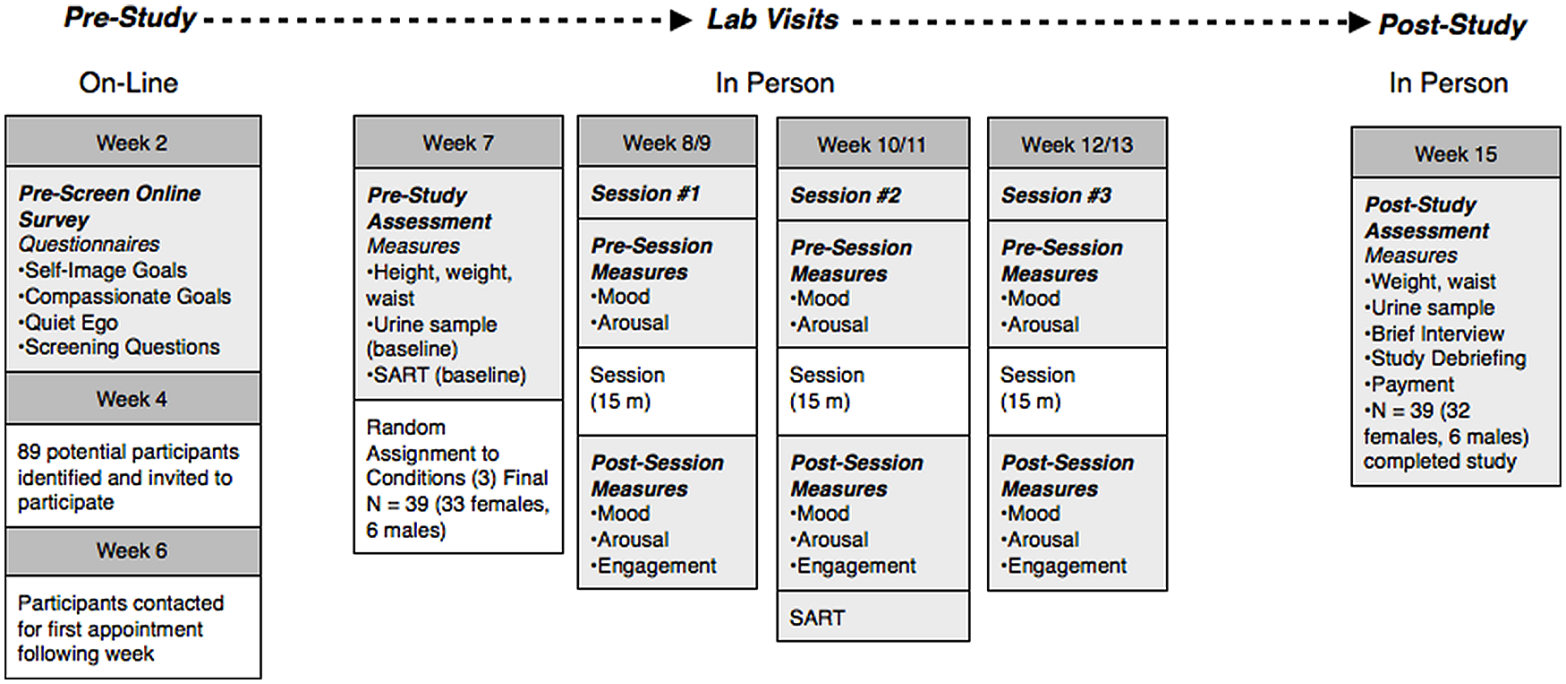
**Study timeline**.

Prior to the start of this study, our experimental protocol was approved by the Northern Arizona University Institutional Review Board. **Figure 1** provides an overview of the procedures involved in this multi-week study: participant recruitment (weeks 2–6), intervention (weeks 7–13), and post-study (weeks 14–15). In order to identify potential study participants, we used results from a questionnaire distributed to all introductory psychology students during the second week of the semester. In that questionnaire, students completed a13-items measure that assessed use of self-image and compassionate interpersonal goals (Crocker and Canevello, 2008). Coefficient alphas were 0.71 and 0.86, respectively. We calculated the ratio of self-image goals to compassionate goals for each student, because students who use self-image goals (and fewer compassionate goals) are at increased risk for self-evaluative stress (Crocker et al., 2010). During the third week of the semester, email invitations were sent to 89 students in their first semester of college. To minimize potential expectancy biases, participants were told that the study was about students’ transition to college and would include the opportunity to learn a method of stress-reduction that may or may not be helpful to them. About half of the invited students (*N* = 42, 49%) responded with interest and were sent a second email providing them with access to an online informed consent form and questionnaire. In order to participate, participants were required to give consent. All subjects gave written informed consent in accordance with the Declaration of Helsinki and the university’s institutional review board. A total of 39 participants (33 female, six male) came to the lab for the first visit. Due to the small number of males (only two per condition) we report on the females in this sample only (*N* = 32).

#### Baseline Visit and Random Assignment to Condition

About the middle of the semester all participants came to the lab for individual appointments and provided anthropomorphic measurements (e.g., changes in BMI could affect oxidative stress measurement), a urine sample, and completed the Sustained Attention to Response Task (SART). At the end of week, all participants were randomly assigned to one of three experimental groups: (1) QEC, (2) QEC with VR headset (QEC-VR), and (3) control group. Participants in the QEC-VR were instructed in how to use the Sony HMZ-T1 VR Headset, which used the WorldViz Vizard V4 platform to display a southern, park-like setting while listening to the identical audio-recorded QEC as participants in the QEC (alone) condition.

#### Intervention Visits

Beginning the week after the baseline visit, each study participant came to the lab three times in the subsequent 4–5 weeks. Participants received a reminder email the evening prior to their visit. The first and third intervention visits took approximately 25 min. The second intervention visit was exactly like the first, with the exception that after the intervention participants returned to the original lab space to complete a cognitive task (SART) increasing the length of the visit to 40 min.

##### Intervention content

Each participant, regardless of the condition, was told that she would be completing a method of stress reduction that was designed to help “think about what is important” and that practicing this method “may make it easier to remember what is important when facing a stressful situation.” All participants were seated in a comfortable chair and asked to turn their cell phones off or to mute. Participants in the QEC-VR condition were given the additional instructions that, prior to the commencement of the QEC, they could become familiar with how to navigate the 3D scene, and that during the QEC they were free to look where they pleased (active navigation). Participants in the control condition were provided with National Geographic magazines to read through and were instructed not to look at other materials, including their cell phones, during their 15-min time. In the two QEC conditions (with and without VR), participants were told that the intervention visit would provide them with an opportunity to be reminded of “well-known ways to cultivate important parts of your identity that will help you become a more balanced person, to keep things in perspective, and remember what may be important to you.” Accordingly, participants in each of the QEC conditions heard a recording that briefly described each of four quiet ego characteristics: detached awareness, inclusive identity, perspective taking, and growth. The text included a definition of the idea along with lay explanation prompted participants to “take a few minutes to think about what the idea meant to them.” For example, in the detached awareness description students heard a description of the importance of “living fully in the present with an open and non-defensive mind.” In the supplementary materials associated with this article we provide a brief overview of these four characteristics as described in the QEC. A copy of the 15-min audio recording can be obtained by contacting the corresponding author. For the control group, we elected a relatively simple and straightforward task: reading *National Geographic* magazines. This publication is known for its award-winning beautiful photos and articles that reflect their mission to inspire care and concern for all living things, including the planet and its inhabitants (people, animals, plants, the environment). We reasoned that looking through these magazines would be conducive to stress reduction. The instructions to the participants in all conditions were very similar: 96% overlap between the QEC and QEC–VR conditions and 84% between QEC and the control conditions.

At the conclusion of the intervention session, all study participants heard “the method of stress reduction that you have just experienced has been designed to help you remember what is important to you. Our recommendation is that you try to remember and use these thoughts and feelings as a way to help you deal with the various tasks and demands facing you. To do that, you may want to mentally rehearse the kinds of thoughts and feelings that you had today while engaging in the activity you did today.”

#### Post-study Lab Visit and Questionnaire

During the last week of the semester participants returned to the lab for their final visit and provided post-study anthropomorphic measurements and urine sample. Participants also were debriefed about the purpose of the entire study and participated in a brief post-study interview. Prior to the last visit, participants completed a very brief online questionnaire (see Self-Report Individual Difference Measures). Participants received course research participation credit and $100.00 for their participation.

#### Self-report Individual Difference Measures

As indicated in **Figure 1**, participants completed questionnaires at different time points during the study. The 14-item Quiet Ego Scale (Wayment et al., 2015) was included in the pre-screening (coefficient alpha = 0.65) and post-study questionnaires (coefficient alpha = 0.67). Pluralistic thinking (Hurtado, 2001) was included in the questionnaire completed just prior to the first lab visit and again in the post-study questionnaire. It is a five-item scale that asked participants to rate themselves accurately on five skills “compared with the average person your age” (ability to see the world from someone else’s perspective, tolerance of others with different beliefs, openness to having my own views challenged, ability to discuss and negotiate controversial issues, and ability to work cooperatively with diverse people). Items were rated on a five-point scale. Coefficient alphas were 0.73 and 0.90 at the two time points (respectively). In the post-study questionnaire participants also answered three items (as a manipulation check) about their recollections of the “stress reduction” sessions. Participants were asked to how “novel” their experience was as well as indicate the extent to which the sessions “helped them be fully present in the moment” and “to imagine beautiful places on earth.” All questions were rated on a five-point scale with appropriately labeled endpoints.

#### Intervention Assessments

Before and after each intervention session participants completed two questions about mood and arousal (Russell et al., 1989) using a visual analog scale (10 cm horizontal line anchored with the endpoints “Extremely unpleasant or negative feelings” and “Extremely pleasant or positive feelings” and “extremely low arousal” to “extremely high arousal”). At the end of each of the 15-min sessions participants were also asked to rate how engaged they were, how enjoyable the experience was, and how useful the intervention was for stress reduction (using a five-point scale with appropriately labeled endpoints). These measures were highly correlated and combined to create an involvement score for each session. Coefficient alphas for the involvement measures were 0.85, 0.74, and 0.90, respectively. We combined these involvement scores into one score representing the total feeling of involvement in the session (enjoyment, usefulness as a stress reduction method) for each participant. After the third session participants were asked how often they had practiced their stress reduction technique in the past week and how much they anticipated using this technique in the future. These two items were correlated and combined to form a practice/intention to practice score.

#### Cognitive and Physiological Measures

The SART is a go/no-go task found to be a valid and useful indicator of mind-wandering (Mrazek et al., 2012b). The time between pre- and post-versions of the test was 3–4 weeks. This behavioral measure of attention was assessed on a desktop computer, using DirectRT v2010 software (Empirisoft Corporation, New York, NY, USA). Participants were asked to respond as quickly as possible to frequent non-targets (instances of letter O appearing on the screen) by pressing the spacebar. Participants were provided with 20 practice trials, followed by 216 non-target and 24 target trials. The total time required to complete the SART was approximately 10 min. Mind-wandering was quantified as the number of errors occurring on the 24 target trials.

Pre- and post-study urine samples (up to 50 cc) were collected during week 7 and week 15 weeks (∼2 months apart). At the time of collection, samples were aliquoted and frozen at -80°C until analysis. Butylated hydroxytoluene was added to the aliquots for 8-iso-PG F2α prior to storing to prevent auto-oxidation. Urinary 8-iso-PG F2α levels were measured using a commercial enzyme immunoassay kit (Cayman Chemical, Ann Arbor, MI, USA). Each sample was analyzed in duplicate at two different dilutions and to minimize analytical variation both samples from each subject were analyzed in the same run. The samples were read at 405 nm in a 96-well plate reader. Urinary 8-iso-PG F2α concentration was adjusted for urinary dilution effects by expressing levels normalized to creatinine excretion. Urinary creatinine was measured using a colorimetric assay kit according to manufacturer’s instructions (Cayman Chemical, Ann Arbor, MI, USA).

### Analysis Strategy

Analyses were conducted with the SPSS 21.0 software package (IBM, Armonk, NY, USA). We were conservative with the number of tests, and used planned comparisons ANOVAs. Where possible, we also report bootstrapped confidence intervals (*N* = 1000). To test our main hypotheses, we followed the advice of Cumming who states that one of the best ways to respond to the “disappointingly large uncertainty revealed by wide CIs” is the small scale meta-analysis from “related results with a single experiment” (Cumming, 2012, p. 184, 186). We report Cohen’s *d* statistics, and 95% confidence intervals, using ESCI (Exploratory Software for Confidence Intervals; www.thenewstatistics.com; Cumming, 2012).

## Results

### Condition Equivalency

**Table 1** presents the baseline measures for participants in the three experimental conditions. Results of a MANOVA on baseline measures found that randomly assigned groups were equivalent on age, quiet ego, and pluralistic thinking, *F*(6,68) = 1.14, *p* = 0.41. A second MANOVA found that the groups were equivalent on the initial measures cognitive focus and oxidative stress, *F*(4,52) = 0.54, *p* = 0.71. We also examined for equivalence on session engagement and number of days in which they completed the three stress reduction sessions, *F*(4,68) = 1.21, *p* = 0.32. Finally, we examined potential differences in ethnic status and parents’ highest levels of education, but could not compute Chi Square analyzes due to cell sizes less than five. Taken together, the three conditions appeared to be equivalent in terms of baseline measures and level of engagement in the sessions.

**Table 1:** Mean (SD) scores for each group on baseline and demographic measures.

	Quiet ego contemplation (QEC) (*n* = 10)	QEC-VR (*n* = 10)	Control (*n* = 12)	*F* (2,29)
**Measures**				
Quiet ego scale	3.44 (0.30) [3.2, 3.7]	3.31 (0.55) [3.1, 3.6]	3.58 (0.32) [3.3, 3.8]	1.22
Pluralistic thinking SART	3.82 (0.91) [3.4, 4.3]	3.62 (0.77) [3.2, 4.1]	3.56 (0.45) [3.4, 4.3]	0.31
	0.86 (0.10) [0.78, 0.94]	0.82 (0.11) [0.73, 0.91]	0.81 (0.15) [0.73, 0.88]	0.47
Oxidative stress	11.05 (7.8) [4.6, 17.5]	8.85 (15.5) [1.6, 16.1]	6.45 (6.09) [0.27, 12.6]	0.56
**Demographics**				
Age	18.10 (0.32) [17.9, 18.3]	18.00 (0.00) [17.8, 18.2]	18.20 (0.41) [18.0, 18.4]	0.96
Involvement^a^	3.72 (0.97)	3.12 (0.80)	3.70 (0.54)	1.17
Days to complete all sessions	29.90 (4.7) [27,32.8]	34.10 (2.9) [31.2, 37]	32.27 (4.7) [29.5, 35.1]	2.20
Caucasian	70%	80%	66%	n/a
Mother attended college	70%	70%	83%	n/a
Father attended college	80%	60%	75%	n/a


*Chi-square analyzes could not be completed because of small cell size (<5).*

*^a^Total involvement score over all three sessions (prior analyzes revealed no time × condition differences for involvement); score is mean of three items: how engaged and how enjoyable, how useful for stress reduction*.

**Table 2** presents participants’ cumulative scores of self-reported mood and arousal ratings before and after their stress reduction experiences. A repeated measures ANOVA on pre- and post-mood by condition revealed that regardless of the condition, mood improved over the course of their 15-min activity sessions, *F*(1,30) = 47.67, *p* < 0.001. Further, there appeared to be no real changes in arousal, regardless of condition. Taken together, across the three conditions, participants appeared to experience a more positive mood, but no real changes in arousal, regardless of their stress reduction intervention.

**Table 2:** Pre- and post-session mood and arousal aggregated across three sessions.

	Mood		Arousal	
		
	Pre-session	Post-session	Pre-session	Post-session
QEC	6.35 (1.4)	7.7 (1.3)	5.02 (1.4)	4.50 (2.4)
QEC–VR	5.04 (1.5)	6.25 (1.6)	3.61 (1.7)	3.68 (2.0)
Control	5.78 (1.3)	7.02 (1.5)	4.32 (2.0)	3.87 (1.7)
Time × Cond *F* (2,29)	0.19		0.62	
Cond *F* (2,29)	2.58^+^		1.0	
Time *F* (1,30)	47.67^∗∗∗^		1.6	


^+^*p* < 0.10, ^∗∗∗^*p* < 0.0001.

### Impact of Condition

In the post-study questionnaire, participants answered items about their study experience. We used planned comparisons to examine differences on three variables. Results are presented in **Table 3**. Participants in both QEC conditions reported their experience as more novel compared to controls (who read *National Geographic* magazines), *t*(28) = 2.73, *p* < 0.01. Participants in the control group were more likely than their counterparts in the QEC sessions to say that their stress reduction sessions were related to imagining beautiful places on earth, *t*(28) = 2.21, *p* < 0.05. QEC participants were more likely to report that their sessions helped them to “be in the moment” compared to QEC–VR and control participants, *t*(28) = 2.66, *p* < 0.05. These results concerning participants’ experiences were confirmed in the exit interviews. Participants in the control group reported that they enjoyed *National Geographic’s* photos and stories about cultures and animals from around the world. Participants in the QEC condition were able to articulate the basic tenets of the QEC, and reported that they particularly enjoyed the mindfulness component. Participants from the QEC–VR condition reported being distracted by the VR headset in that it was somewhat uncomfortable and navigating the 3D image appeared to reduce their ability to remember the content of the QEC. Thus, contrary to our expectation, rather than augmenting the QEC, adding the VR to the QEC reduced the effectiveness of the QEC message. To test our final hypotheses, we examined the pre- post-differences for each group separately using paired comparison *t*-tests. We expected hypothesized changes for the QEC group only.

**Table 3:** Mean (SD) scores for each group on manipulation check items.

	QEC	QEC-VR	Control	*t*(28)
Novelty	3.70 (0.95)^a^	3.80 (1.2)^a^	2.72 (0.79)^b^	2.73^∗∗^
“In the moment”	4.20 (0.85)^a^	3.30 (1.1)^b^	3.58 (0.96)^b^	2.66^∗^
“World is beautiful”	3.20 (1.5)^b^	3.40 (0.97)^b^	4.18 (0.60)^a^	2.21^∗^
Practice/intention to practice	2.85 (1.7)^a^	1.7 (1.2)^b^	2.00 (0.77)^b^	2.07^∗^


*^∗^p < 0.05, ^∗∗^p < 0.01. Mean separated by a different superscript (e.g., a, b) were significantly different p < 0.05.*

Presented in percent change from baseline. Mean ± SEM.

### Hypothesis Testing

**FIGURE 2:**
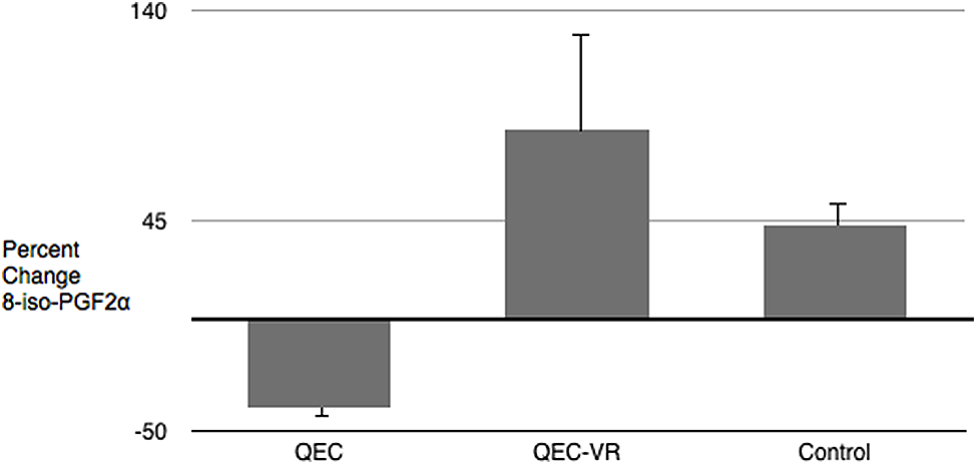
Pre- and post-study change in oxidative stress (8-iso-PGF2α).

**FIGURE 3:**
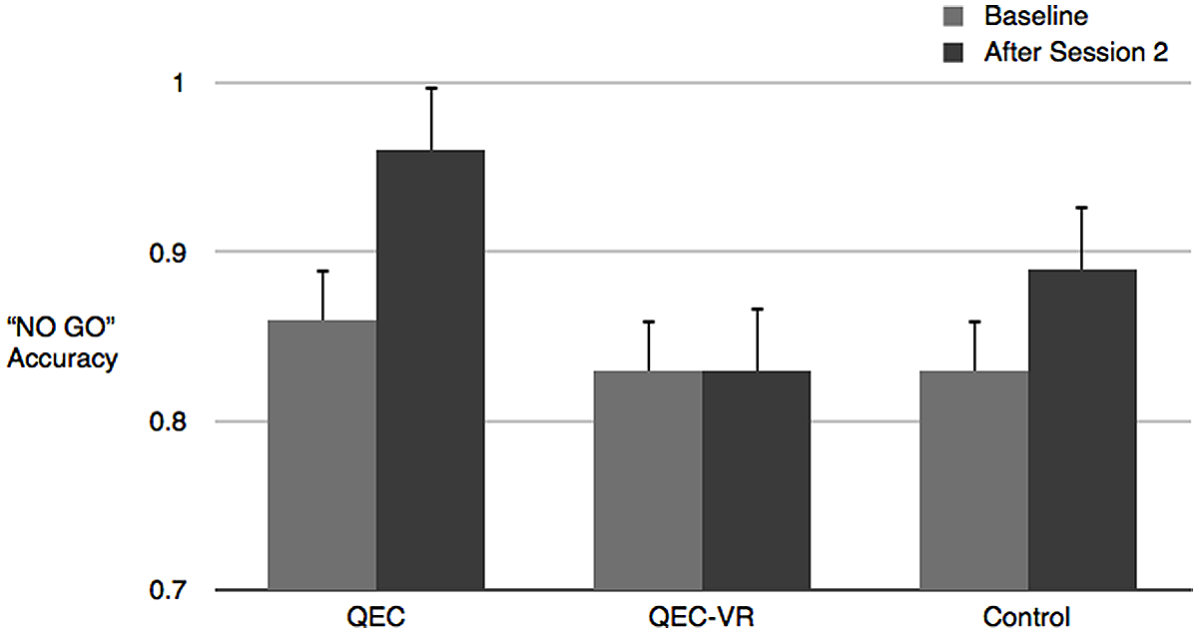
**Pre- and post-scores on mind-wandering.** Mean ± SEM.

To examine whether the QEC had the intended effects on strengthening a compassionate self-identity paired *t*-tests and effect sizes were computed for each experimental condition on measures of quiet ego and pluralistic thinking. In the QEC condition, quiet ego characteristics and pluralistic thinking increased (albeit modestly) over time, in line with expectations. There were no changes in the QEC–VR or control conditions. In line with our expectations, paired *t*-tests revealed a moderate decrease in oxidative stress for QEC group, but no change for the QEC–VR or control participants. See **Figure 2**. For mind-wandering, there were improvements for both the QEC and control groups, but not for those in the QEC–VR condition (see **Figure 3**). Group means, standard deviations, 95% confidence interval bootstrapped estimates are listed in **Table 4**. Given our small sample size, we also converted the Cohen’s *d* statistics to Hedge’s *g* statistics. Using Cumming’s Excel-based ESCI program (2012), we created separate tree plots for each experimental group. Results revealed a moderate overall effect for the QEC group, Cohen’s *d* = 0.64 [0.29, 0.98], unbiased *d* = 0.58 [0.27, 0.89]. There was no overall effect for QEC combined with a VR forest scene, Cohen’s *d* = –0.04 [–0.40, 0.27], unbiased *d* = –0.04 [–0.32, 0.25] or for the National Geographic control, Cohen’s *d* = 0.01 [–0.39, 0.42], unbiased *d* = 0.01 [–0.36, 0.38]. On average, the audio QEC was associated with changes equivalent to about one-half standard deviation in relevant outcomes.

**Table 4:** Pre–post changes on outcome variables: paired *t*-tests, effect sizes, and 95% CIs.

	Pre 95% CI	Post 95% CI	*t*_paired_	*d*_Cohen_
**QEC condition**				
Quiet ego	3.44 (0.30) [3.3, 3.6]	3.61 (0.27) [3.5, 3.8]	1.46	0.46
Pluralistic thinking	3.82 (0.91) [3.3, 4.4]	4.00 (0.69) [3.6,4.4]	1.37	0.48
SART	0.86 (0.10) [0.79, 0.91	0.96 (0.04) [0.93, 0.98]	2.80	0.94
Oxidative stress	11.88 (7.8) [7.3, 17.0]	7.04 (6.1) [3.5, 11.1]	2.20	0.75
Average effect size	*d* = 0.64 [0.29, 0.98], unbiased *d* = 0.58 [0.27, 0.89]			
**QEC-VR condition**				
Quiet ego	3.31 (0.55) [3.0, 3.6]	3.23 (0.30) [3.1, 3.4]	–0.62	–0.25
Pluralistic thinking	3.62 (0.77) [3.2, 4.1]	3.60 (0.66) [3.2, 4.0]	0.17	0.05
SART	0.83 (0.10) [0.77, 0.89]	0.83 (0.16) [0.73, 0.91]	0.11	0.04
Oxidative stress	8.85 (15.5) [2.7, 20.4]	8.88 (9.8) [3.8, 16.2]	–0.01	–0.01
Average effect size	*d* = –0.04 [–0.40, 0.27], unbiased *d* = –0.04 [–0.32, 0.25]			
**Control condition**				
Quiet ego	3.58 (0.32) [3.4, 3.8]	3.62 (0.34) [3.4, 3.8]	0.35	0.11
Pluralistic thinking	3.85 (0.45) [3.6, 4.1]	3.78 (0.60) [3.5, 4.1]	–0.57	–0.18
SART	0.80 (0.14) [0.72, 0.89]	0.87 (0.11) [0.85, 0.95]	1.99	0.56
Oxidative stress	6.45 (6.4) [3.2, 10.8]	7.35 (7.2) [4.1, 12.3]	–1.3	–0.41
Average effect size	*d* = 0.01 [–0.39, 0.42], unbiased *d* = 0.01 [–0.36, 0.38]			


## Discussion

Our study participants were in their first semester of college, a time characterized by self-evaluative threat (Wayment and Taylor, 1995) and a time where self-integrity may be threatened (Cohen and Sherman, 2014). We tested a novel intervention that, unlike more complex and time-consuming stress reduction methods (Regehr et al., 2013), is brief and could be self-administered. Our results (including exit interviews) suggested that students who were reminded of transcendent values understood those values and found the intervention useful to reduce stress. These students also reported having practiced the technique on their own and that they intended to use the ideas in the future to help them cope with stress. Women in this group reported higher quiet ego characteristic scores and more pluralistic thinking over time. Thus, we have some initial evidence that the QEC recording that we developed was useful in reminding students about the quiet ego characteristics and may have had the intended effect of balancing self- and other-concerns.

We also found that listening to the brief QEC in the lab significantly reduced mind-wandering. If we assume that the transition to college can generate negative emotional states, then the work of Smallwood et al. (2009) has relevance. Smallwood et al. (2009) suspect that negative emotional experiences may prime the individual to mind-wander and they have been among the first to establish a causal role of mood in attentional lapses. These researchers have induced positive, negative or neutral moods prior to a sustained attention task and found, in particular, that negative mood reduces commitment to the task at hand and increases focus on task irrelevant personal concerns. Others have shown that mindfulness interventions can reduce mind-wandering or intrusive thoughts cued by exposure to material associated with stressful events (Nassif and Wells, 2014). Thus, to the extent that being reminded about one’s quiet ego characteristics can help focus attention on task-relevant concerns, rather than intrusive thoughts that commonly accompany stress, the QEC may help to reduce mind-wandering.

The brief QEC intervention also significantly reduced levels of oxidative stress. Previous studies on meditation, yoga, diaphragmatic breathing and other mind-body practices have found lowering of oxidative stress and inflammatory markers (Yadav et al., 2005; Martarelli et al., 2011; Bhasin et al., 2013; Lavretsky et al., 2013; Bower et al., 2015). These studies also tested very short interventions (e.g., 12–15 min), but had a greater number of sessions than employed in the present study. Participants in our study were asked how often they thought they would practice the stress reduction technique they learned, and those in the QEC condition reported the highest scores on practice/intention to practice in the future. Although these were self-reports, it may be that if students thought about the QEC prime outside of their intervention session, this practice may have helped to interrupt pathways involved in oxidative stress. Although the literature on oxidative stress suggests an association between altered antioxidant capacity and psychiatric disorders, including psychological or emotional distress (see Salim, 2014 for review), the causal relationship is not fully understood. Our findings should be replicated in a larger study and should include additional markers of oxidative stress and inflammation.

Taken together, an examination of our very brief QEC intervention appeared to help reduce mind-wandering and oxidative stress. Our results add to the growing literature on the benefits of very brief interventions. In a recent study, a 10-min loving kindness intervention increased compassion, decreased self-focus, and increased feeling a sense of connection (Seppala et al., 2014). Our results also add to the self-affirmation literature in that the QEC reminds students of self-transcendent values (Crocker et al., 2008; Shnabel et al., 2013). Self-transcendent affirmations have been shown to be beneficial in a variety of contexts, including education and health (Cohen and Sherman, 2014), and when delivered prior to the initiation of a defensive response (Critcher et al., 2010).

We added a VR condition to our study because we were interested in examining if being immersed in a 3D nature scene would enhance participants’ focus and intensify the experience. However, we were surprised to find that our findings QEC–VR condition were contrary to prediction; in fact, by adding the VR component, the outcomes were actually *worse* than in the control condition. In most of the VR literature published to date, the addition of VR appears to augment wellness interventions, not detract from them, with VR interventions showing large effect sizes compared to non-interventions and moderate effect sizes compared to active interventions (e.g., Turner and Casey, 2014). One reason for our results may be due to the lower quality VR headset that we used; it was uncomfortable for most participants. In addition, while participants could physically stand and move in the virtual park (360°), their movements were self-directed. It may be that a guided or more coordinated (e.g., passive) experience may be less distracting. For example, when listening to specific parts of the QEC, the viewer could be guided through a beautiful scene, with timed stops to better hear the recording. Hence, it may be that participants’ self-navigation through the scene could have been a distraction from the QEC. Investigators using VR should consider integrating the VR with the audio for better outcomes, providing specific cues and directions for viewing, and/or a way to track participants’ exploration in the VR environment. Quite possibly there are many more VR studies with *negative* results that are never published. Although our results may be cautionary, advances in technology and visual aids (e.g., Oculus Rift, the latest in VR stereoscopic head-mounted displays), suggest that future research should continue to examine the potential benefit of technology that could, when combined with other interventions, improve their effectiveness in a clinical setting.

Additional areas for future research include elucidating the mechanisms by which a very brief reminder of quiet ego characteristics may be beneficial. In our study, the beneficial effects associated with the QEC were not due to improvements in mood; respondents in all three conditions reported an overall elevation in mood following their 15-min exercise. The effect may also not have been due necessarily to a decrease in arousal. Both the QEC and the *National Geographic* control group reported modest decreases in arousal. Instead, our preliminary results suggest that reminding individuals of quiet ego characteristics is helpful because of the ability to reduce ruminative self-focus and enhance a reflective focus on the “self” (Watkins, 2008). Most recently, our research has focused on this question. In several first-year courses, we created an extra credit web-exercise where students could listen to a 10-min (or 4 min) QEC and then complete a brief exercise about one of the four quiet ego characteristics. For each of four online exercises, students reported that their thought processes during the QEC were more reflective in nature, and not ruminative (Wayment and Collier, unpublished data). Future research might also examine more closely the effect of a brief QEC intervention on reducing the frequency of intrusive thought (Nassif and Wells, 2014). Another likely mechanism as to why a more compassionate self-identity is helpful in reducing stress is the ability to show the self more compassion (Leary et al., 2007). Quiet ego has not only been found to be moderately associated with self-compassion, but self-compassion mediates the relationship between quiet ego and perceived stress in college students (Wayment et al., in press) and between quiet ego and self-reported health in unemployed adults (Wayment et al., under review).

Future research should also consider how the temporal placement of the QEC might increase its effectiveness for promoting longer-term change (Critcher et al., 2010; Cohen and Sherman, 2014). For example, could a QEC be most helpful after a stressful event has occurred or as a preventive resource, or both? Studies that incorporate supportive mechanisms to remind individuals about affirming self-expansive values have shown success (Armitage et al., 2011). At the end of the study reported here we provided all participants with a cue card containing a brief mnemonic of the four quiet ego characteristics (A–B–C–D) to help them remember these key characteristics. We have included a copy of this card in the supplementary materials. This card can be put in a wallet or in a place where the reminder could prompt a less defensive response to a challenging circumstance (Critcher et al., 2010). Such a reminder could complement more in-depth interventions and activities that are known to help individuals learn to cope more effectively with stress, such as in a work environment (Huffman et al., in press).

### Limitations

Our sample was small. However, we increased our power because we tested *a priori* hypotheses using planned comparisons. We also found moderate effect sizes in the expected direction. It is our hope that our results will be able to be incorporated into meta-analytic studies that address the impact of very brief cognitive interventions on physiological markers of stress and mind-wandering. The gender composition of our sample (female) was also a limitation and therefore our results do not generalize to male college students or to older adults or children. Future studies should examine the QEC in diverse samples to better understand its potential effectiveness in more diverse groups. Another limitation in our findings concerns whether other factors could have influenced the change in F2-isoprostanes over time. Although our findings controlled for potential changes in participant body weight and girth, other factors should be considered, including levels of fitness and in women, menstrual cycle. Traustadottir has found that menstrual cycle does not have any effect on oxidative stress in a sample of pre-menopausal college-aged women, specifically F2-isoprostanes and levels of fitness are less of an issue in samples of young adults (Traustadottir, unpublished data). Finally, although unintended, for participants in our VR condition, the VR experience appeared to interfere with the message of the QEC. Yet, there is promising work regarding the health effects of visual imagery of nature (Selub and Logan, 2012). Future work integrating quiet ego and awe research questions will benefit from more careful consideration of how to incorporate reminding individuals of quiet ego characteristics while drawing on the potentially powerful impact of nature to increase a sense of awareness (Berman et al., 2008), empathy (Weinstein, 2009), connected to nature and living things (Cervinka et al., 2012), and well-being by reducing stress (van der Berg, 2010). Although not the main focus of our study, participants whose experience included reading *National Geographic* magazine, with its focus on nature and wildlife, was favorably received by students and had positive effects on reducing mind-wandering. Future work might include stress reduction techniques that utilize the QEC intervention recording while engaging with nature (e.g., walking or sitting in a natural setting). Strengths of the study design included its longitudinal design, novel content of intervention material, use of a computerized test of mind-wandering, and a measure of oxidative stress to assess physiological impact. Our results, although modest, lend support to a growing literature on the importance of self-expansive and compassionate values.

## Conclusion

We examined a brief intervention to remind individuals of important values associated with an expanded self-identity, a self-identity characterized by balance and growth, a compassionate self-identity that balances concern for self and others (Bauer and Wayment, 2008). Our results offer modest evidence that a brief intervention based on these ideas was effective. Over time, those who had listened to the QEC showed decreases in mind-wandering on a cognitive task and decreases in oxidative stress, a marker of physiological stress. Our work conceptually replicates research on the stress-reducing benefits of affirming self-expansive values. Our results also extend the literature on the benefits of quieting the ego (Bauer and Wayment, 2008). The quiet ego concept represents ideas from long-standing humanistic, philosophical and spiritual traditions related to eudemonic well-being. In fact, a recently proposed humanistic model of spiritual growth (Kass, 2015) suggests five pathways that closely resemble the quiet ego characteristics: behavioral self-regulation through mindfulness, cognitive understanding of human suffering that supports social justice, social–emotional development of compassion toward the self and others, and a resilient worldview and confidence in self to mature in the face of life’s difficulties. Kass’ model also specifies a fifth pathway of contemplative practice. The QEC is a brief intervention that reinforces important values and skills that supports an ability to see the self and others more clearly and less defensively. The benefits of such a brief intervention could be important in offering students an accessible cognitive strategy for what Lukianoff and Haidt (2015) discuss as types of distorted thinking associated with stress, depression, and anxiety common among today’s college students. Taken together, our results add to the literature on the importance of considering the role of excessive self-focus in stress interventions and that interventions that can expand the sense of self may be effective (cf. Cohen and Sherman, 2014).

## Conflict of Interest Statement

The authors declare that the research was conducted in the absence of any commercial or financial relationships that could be construed as a potential conflict of interest.

## References

[B1] Abelson J. L. Erickson T. M. Mayer S. E. Crocker J. Briggs H. Lopez-Duran N. L. (2014). Brief cognitive intervention can modulate neuroendocrine stress responses to the Trier Social Stress Test: buffering effects of a compassionate goal orientation. *Psychoneuroendocrinology* 44 60–70. 10.1016/j.psyneuen.2014.02.016 24767620 PMC4120861

[B2] ACHA (2014). *American College Health Association-National College Health Assessment II: Reference Group Executive Summary Spring 2014*. Hanover, MD: ACHA.

[B3] Armitage C. J. Harris P. R. Arden M. A. (2011). Evidence that self-affirmation reduces alcohol consumption: randomized exploratory trial with a new, brief means of self- affirming. *Health Psychol.* 30 633–641. 10.1037/a0023738 21553966

[B4] Banks J. B. Welhaf M. S. Srour A. (2015). The protective effects of brief mindfulness meditation training. *Conscious. Cogn.* 33 277–285. 10.1016/j.concog.2015.01.016 25680006

[B5] Baños R. M. Guillen V. Quero S. García-Palacios A. Alcaniz M. Botella C. (2011). A virtual reality system for the treatment of stress-related disorders: a preliminary analysis of efficacy compared to a standard cognitive behavioral program. *Inter. J. Human Comput. Stud.* 69 602–613. 10.1016/j.ijhcs.2011.06.002

[B6] Bauer J. J. Wayment H. A. (2008). “The psychology of quieting the ego,” in *Transcending Self-Interest: Psychological Explorations of the Quiet Ego*, eds Heidi J. J. B. Wayment A. (Washington, DC: APA Books), 7–19.

[B7] Berman M. G. Jonides J. Kaplan S. (2008). The cognitive benefits of interacting with nature. *Psychol. Sci.* 19 1207–1212. 10.1111/j.1467-9280.2008.02225.x 19121124

[B8] Bhasin M. K. Dusek J. A. Chang B.-H. Joseph M. G. Denninger J. W. Fricchione G. L. (2013). Relaxation response induces temporal transcriptome changes in energy metabolism. Insulin secretion and inflammatory pathways. *PLoS ONE* 8:e62817 10.1371/journal.pone.0062817 PMC3641112 23650531

[B9] Bower J. E. Crosswell A. D. Stanton A. L. Crespi C. M. Winston D. Arevalo J. (2015). Mindfulness meditation for younger breast cancer survivors: a randomized controlled trial. *Cancer* 121 1231–1240. 10.1002/cncr.29194 25537522 PMC4393338

[B10] Burson A. Crocker J. Mischkowski D. (2012). Two types of value affirmation: implications for self-control following social exclusion. *Soc. Psychol. Pers. Sci.* 3 510–516. 10.1177/1948550611427773

[B11] Canevello A. Crocker J. (2011). Changing relationship growth belief: intrapersonal and interpersonal consequences of compassionate goals. *Pers. Relatsh.* 18 370–391. 10.1111/j.1475-6811.2010.01296.x 21949478 PMC3176594

[B12] Cervinka R. Röderer K. Hefler E. (2012). Are nature lovers happy? On various indicators of well-being and connectedness with nature. *J. Health Psychol.* 17 379–388. 10.1177/1359105311416873 21859800

[B13] Chiesa A. Calati R. Serretti A. (2011). Does mindfulness training improve cognitive abilities? A systematic review of neuropsychological findings. *Clin. Psychol. Rev.* 31 449–464. 10.1016/j.cpr.2010.11.003 21183265

[B14] Cohen G. L. Sherman D. K. (2014). The psychology of change: self-affirmation and social psychological intervention. *Annu. Rev. Psychol.* 65 333–371. 10.1146/annurev-psych-010213-115137 24405362

[B15] Correll J. Spencer S. J. Zanna M. P. (2004). An affirmed self and an open mind: self- affirmation and sensitivity to argument strength. *J. Exp. Soc. Psychol.* 40 350–356. 10.1016/j.jesp.2003.07.001

[B16] Creswell J. D. Welch W. T. Taylor S. E. Sherman D. K. Gruenewald T. L. Mann T. (2005). Affirmation of personal values buffers neuroendocrine and psychological stress responses. *Psychol. Sci.* 16 846–851. 10.1111/j.1467-9280.2005.01624.x 16262767

[B17] Critcher C. R. Dunning D. (2015). Self-affirmations provide a broader perspective on self- threat. *Pers. Soc. Psychol. Bull.* 41 3–18. 10.1177/0146167214554956 25319717

[B18] Critcher C. R. Dunning D. Armor D. A. (2010). When self-affirmations reduce defensiveness: timing is key. *Pers. Soc. Psychol. Bull.* 36 947–959. 10.1177/0146167210369557 20505163

[B19] Crocker J. Canevello A. (2008). Creating and undermining social support in communal relationships: the role of compassionate and self-image goals. *J. Pers. Soc. Psychol.* 95 555–575. 10.1037/0022-3514.95.3.555 18729694

[B20] Crocker J. Canevello A. Breines J. G. Flynn H. (2010). Interpersonal goals and change in anxiety and dysphoria in first-semester college students. *J. Pers. Soc. Psychol.* 98 1009–1024. 10.1037/a0019400 20515255 PMC2966869

[B21] Crocker J. Niiya Y. Mischkowski D. (2008). Why does writing about important values reduce defensiveness? Self-affirmation and the role of positive other-directed feelings. *Psychol. Sci.* 19 740–747. 10.1111/j.1467-9280.2008.02150.x 18727791

[B22] Cumming G. (2012). *Understanding the New Statistics: Effect Sizes, Confidence Intervals, and Meta-Analysis*. New York, NY: Routledge.

[B23] Dyson R. Renk K. (2006). Freshmen adaptation to university life: depressive symptoms, stress, and coping. *J. Clin. Psychol.* 62 1231–1244. 10.1002/jclp.20295 16810671

[B24] Ghanbarzadeh R. Ghapanchi A. H. Blumenstein M. Talaei-Khoei A. (2014). A decade of research on the use of three-dimensional virtual worlds in health care: a systematic literature review. *J. Med. Int. Res.* 16:e47 10.2196/jmir.3097 PMC3958677 24550130

[B25] Gilbert P. (2009). Introducing compassion-focused therapy. *Adv. Psychol. Treat.* 15 199–208. 10.1002/cpp.1806

[B26] Gilbert P. Procter S. (2006). Compassionate mind training for people with high shame and self-criticism: overview and pilot study of a group therapy approach. *Clin. Psychol. Psychother.* 13 353–379. 10.1002/cpp.507

[B27] Gingrich J. A. (2005). Oxidative stress is the new stress. *Nat. Med.* 11 1281–1282. 10.1038/nm1205-1281 16333265

[B28] Greenberg J. Pyszczynski T. (1986). Persistent high self-focus after failure and low self- focus after success: the depressive self-focusing style. *J. Pers. Soc. Psychol.* 50 1039–1044. 10.1037/0022-3514.50.5.1039 3712228

[B29] Helgeson V. S. (1994). Relation of agency and communion to well-being: evidence and potential explanations. *Psychol. Bull.* 116 412–428. 10.1037/0033-2909.116.3.412

[B30] Huffman A. H. Irving L. Wayment W. A. (in press). The quiet ego: assuaging organizational concerns about mindfulness. *Ind. Organ. Psychol.*

[B31] Hurtado S. (2001). *Linking Diversity and Educational Purpose [microform]: How Diversity Affects the Classroom Environment and Student Development / Sylvia Hurtado*. Washington, DC: Distributed by ERIC Clearinghouse.

[B32] Kass J. D. (2015). Person-centered spiritual maturation: a multidimensional model. *J. Hum. Psychol.* 55 53–76. 10.1177/0022167814525261

[B33] Klimecki O. M. Leiberg S. Lamm C. Singer T. (2013). Functional neural plasticity and associated changes in positive affect after compassion training. *Cereb. Cortex* 23 1552–1561. 10.1093/cercor/bhs142 22661409

[B34] Lavretsky H. Epel E. S. Siddarth P. Nazarian N. Cyr N. S. Khalsa D. S. (2013). A pilot study of yogic meditation for family dementia caregivers with depressive symptoms: effects on mental health, cognition, and telomerase activity. *Int. J. Geriatr. Psychiatry* 28 57–65. 10.1002/gps.3790 22407663 PMC3423469

[B35] Leary M. R. (2004). *The Curse of the Self: Self-Awareness, Egotism, and the Quality of Life*. Oxford: Oxford University.

[B36] Leary M. R. Tate E. B. Adams C. E. Allen A. B. Hancock J. (2007). Self-compassion and reactions to unpleasant self-relevant events: the implications of treating oneself kindly. *J. Pers. Soc. Psychol.* 92 887–904. 10.1037/0022-3514.92.5.887 17484611

[B37] Lejuez C. W. Hopko D. R. Hopko S. D. (2001). A brief behavioral activation treatment for depression. *Treat. Manual. Behav. Modif.* 25 255–286. 10.1177/0145445501252005 11317637

[B38] Logel C. Cohen G. L. (2012). The role of the self in physical health: testing the effect of a values-affirmation intervention on weight loss. *Psychol. Sci.* 23 53–55. 10.1177/0956797611421936 22157517

[B39] Lukianoff G. Haidt J. (2015). The coddling of the American mind. *Atlantic* 316 42–53.

[B40] Martarelli D. Cocchioni M. Scuri S. Pompei P. (2011). Diaphragmatic breathing reduces exercise-induced oxidative stress. *Evid. Based Complement Alternat. Med.* 2011 932430 10.1093/ecam/nep169 PMC3139518 19875429

[B41] Milne G. L. Musiek E. S. Morrow J. D. (2005). F2-isoprostanes as markers of oxidative stress in vivo: an overview. *Biomarkers* 10(Suppl. 1), S10–S23. 10.1080/13547500500216546 16298907

[B42] Mor N. Winquist J. (2002). Self-focused attention and negative affect: a meta-analysis. *Psychol. Bull.* 128 638–662. 10.1037/0033-2909.128.4.638 12081086

[B43] Mrazek M. D. Smallwood J. Franklin M. S. Chin J. M. Baird B. Schooler J. W. (2012a). The role of mind-wandering in measurements of general aptitude. *J. Exp. Psychol. Gen.* 141 788–798. 10.1037/a0027968 22468669

[B44] Mrazek M. D. Smallwood J. Schooler J. W. (2012b). Mindfulness and mind-wandering: finding convergence through opposing constructs. *Emotion* 12 442–448. 10.1037/a0026678 22309719

[B45] Nassif Y. Wells A. (2014). Attention training reduces intrusive thoughts cued by a narrative of stressful life events: a controlled study. *J. Clin. Psychol.* 70 510–517. 10.1002/jclp.22047 24114746

[B46] Palta P. Samuel L. J. Miller E. R. III Szanton S. L. (2014). Depression and oxidative stress: results from a meta-analysis of observational studies. *Psychosom. Med.* 76 12–19. 10.1097/PSY.0000000000000009 24336428 PMC4290164

[B47] Pryor J. H. Hurtado S. DeAngelo L. Paluki Blake L. Tran S. (2010). *The American Freshman: National Norms Fall 2010*. Los Angeles, CA: Higher Education Research Institute, UCLA.

[B48] Regehr C. Glancy D. Pitts A. (2013). Interventions to reduce stress in university students: a review and meta-analysis. *J. Affect. Disord.* 148 1–11. 10.1016/j.jad.2012.11.026 23246209

[B49] Riva G. (2005). Virtual reality in psychotherapy: review. *Cyberpsychol. Behav.* 8 220–230. 10.1089/cpb.2005.8.220 15971972

[B50] Riva G. Bacchetta M. Baruffi M. Rinaldi S. Vincelli F. Molinari E. (2000). Virtual reality-based experiential cognitive treatment of obesity and binge-eating disorders. *Clin. Psychol. Psychother.* 7 209–219. 10.1002/1099-0879(200007)7:3<209::AID-CPP232>3.0.CO;2-V

[B51] Riva G. Baños R. Botella C. Wiederhold B. K. Gaggioli A. (2012). Positive technology: using interactive technologies to promote positive functioning. *Cyber Psychol. Behav. Soc. Netw.* 15 697–724. 10.1089/cyber.2011.0139 22149077

[B52] Roeser R. W. Pinela C. (2014). Mindfulness and compassion training in adolescence: a developmental contemplative science perspective. *New Dir. Youth Dev.* 142 9–30. 10.1002/yd.20094 25100492

[B53] Ross S. E. Neibling B. C. Heckert T. M. (1999). Sources of stress among college students. *Coll. Stud. J.* 33 312–317.

[B54] Russell J. A. Weiss A. Mendelsohn G. A. (1989). Affect Grid: a single-item scale of pleasure and arousal. *J. Person Soc. Psychol.* 57 493–502. 10.1037/0022-3514.57.3.493

[B55] Salim S. (2014). Oxidative stress and psychological disorders. *Curr. Neuropharm.* 12 140–147. 10.2174/1570159X11666131120230309 PMC3964745 24669208

[B56] Schimel J. Arndt J. Banko K. M. Cook A. (2004). Not all self-affirmations were created equal: the cognitive and social benefits of affirming the intrinsic (vs. extrinsic) self. *Soc. Cogn.* 22 75–99. 10.1521/soco.22.1.75.30984

[B57] Selub E. Logan A. (2012). *Your Brain on Nature.* Mississauga, ON: Wiley.

[B58] Seppala E. Hutcherson C. Nguyen D. Doty J. Gross J. (2014). Loving-kindness meditation: a tool to improve healthcare provider compassion, resilience, and patient care. *J. Compass Health Care* 1 5 10.1186/s40639-014-0005-9

[B59] Sherman D. K. Bunyan D. P. Creswell J. D. Jaremka L. M. (2009). Psychological vulnerability and stress: the effects of self-affirmation on sympathetic nervous system responses to naturalistic stressors. *Health Psychol.* 28 554–562. 10.1037/a0014663 19751081

[B60] Shiota M. N. Campos B. Keltner D. (2003). The faces of positive emotion: prototype displays of awe, amusement, and pride. *Ann. N. Y. Acad. Sci.* 1000 296–299. 10.1196/annals.1280.029 14766641

[B61] Shnabel N. Purdie-Vaughns V. Cook J. E. Garcia J. Cohen G. L. (2013). Demystifying values-affirmation interventions: writing about social belonging is a key to buffering against identity threat. *Pers. Soc. Psychol. Bull.* 39 663–676. 10.1177/0146167213480816 23478675

[B62] Smallwood J. Fitzgerald A. Miles L. K. Phillips L. H. (2009). Shifting moods, wandering minds: negative moods lead the mind to wander. *Emotion* 9 271–276. 10.1037/a0014855 19348539

[B63] Traustadottir T. Davies S. S. Su Y. Choi L. Brown-Borg H. M. Roberts L. J. (2012). Oxidative stress in older adults: effects of physical fitness. *Age (Dordr)* 34 969–982. 10.1007/s11357-011-9277-6 21671197 PMC3682074

[B64] Turner W. A. Casey L. M. (2014). Outcomes associated with virtual reality in psychological interventions: where are we now? *Clin. Psychol. Rev.* 34 634–644. 10.1016/j.cpr.2014.10.003 25455627

[B65] Valtchanov D. Barton K. R. Ellard C. (2010). Restorative effects of virtual nature settings. *Cyberpsychol. Behav. Soc. Netw.* 13 503–512. 10.1089/cyber.2009.0308 20950174

[B66] van der Berg A. (2010). Green space as a buffer between stressful life events and health. *Soc. Sci. Med.* 70 1203–1210. 10.1016/j.socscimed.2010.01.002 20163905

[B67] Villani D. Riva G. (2012). Does interactive media enhance the management of stress? Suggestions from a controlled study. *Cyberpsychol. Behav. Soc. Netw.* 15 24–30. 10.1089/cyber.2011.0141 22032797

[B68] Watkins E. (2008). Constructive and unconstructive repetitive thought. *Psychol. Bull.* 134 163–206. 10.1037/0033-2909.134.2.163 18298268 PMC2672052

[B69] Wayment H. A. Bauer J. (eds). (2008). *Transcending Self-Interest: Psychological Explorations of the Quiet Ego*. Washington, DC: American Psychological Association.

[B70] Wayment H. A. Bauer J. J. Sylaska K. (2015). The quiet ego scale: measuring the Compassionate Self-Identity. *J. Happiness Stud.* 16 999–1033. 10.1007/s10902-014-9546-z

[B71] Wayment H. A. Taylor S. E. (1995). Self-evaluation processes: motives, information use, and self-esteem. *J. Pers.* 63 729–757. 10.1111/j.1467-6494.1995.tb00315.x 8531044

[B72] Wayment H. A. West T. Craddock E. (in press). Compassionate values as a resource during the transition to college: quiet ego, compassionate goals, and self-compassion. *J. First Year Exp. Stud. Transit.*

[B73] Weinstein N. (2009). Can nature make us more caring? Effects of immersion in nature on intrinsic aspirations and generosity. *Pers. Soc. Psychol. Bull.* 35 1315 10.1177/0146167209341649 19657048

[B74] Wood J. V. Saltzberg J. A. Neale J. M. Stone A. A. Rachmiel T. B. (1990). Self-focused attention, coping responses, and distressed mood in everyday life. *J. Pers. Soc. Psychol.* 58 1027–1036. 10.1037/0022-3514.58.6.1027 2391637

[B75] Yadav R. K. Ray R. B. Vempati R. Bijlani R. L. (2005). Effect of a comprehensive yoga- based lifestyle modification program on lipid peroxidation. *Indian J. Physiol. Pharmacol.* 49 358–362. 16440857

[B76] Zeidan F. Johnson S. K. Diamond B. J. David Z. Goolkasian P. (2010). Mindfulness meditation improves cognition: evidence of brief mental training. *Conscious. Cogn.* 19 597–605. 10.1016/j.concog.2010.03.014 20363650

