# Supplementary material for: Brief quiet ego contemplation reduces oxidative stress and mind-wandering
Source: Front Psychol. 2015 Sep 30;6:1481. doi: 10.3389/fpsyg.2015.01481 (PMC4588101; doi:10.3389/fpsyg.2015.01481)
Supplement: Supplementary file 1 [file Image_1.PDF]

# What Quiets the Ego?

Be here **now**, in the present moment, without judging yourself or others. Be **open-minded.**
(This is called Detached Awareness)

Think about what it means to be human and what you share with all living things. Remember that we are all **interconnected**.
(This is called Inclusive Identity)

Be open to different perspectives; this cultivates empathy and compassion. Be **open-hearted**, and give others the benefit of the doubt. (This is called Perspective Taking)

Live a valuable and **meaningful** life; strive to **grow** and **learn** from your successes and mistakes.
(This is called Growth)

## *Quiet Ego Contemplation*

### Awareness
*without judgment of self and others*

### Belonging
*we are connected to others and to the natural world*

### Compassion
*see other perspectives; identify with others; be open-hearted*

### Development
*learn from your mistakes, become a more balanced person*

NORTHERN ARIZONA UNIVERSITY

© Heidi Wayment

Psychological Sciences
Box 15106
Flagstaff, Arizona 86011
